# Management of Multiple Myeloma: A Review for General Practitioners in Oncology

**DOI:** 10.3390/curroncol30050334

**Published:** 2023-04-22

**Authors:** Bethany E. Monteith, Irwindeep Sandhu, Ann S. Lee

**Affiliations:** 1 Department of Medicine, Queen’s University, Kingston, ON K7L 3N6, Canada; bethany.monteith@kingstonhsc.ca; 2 Department of Oncology, Cross Cancer Institute, University of Alberta, Edmonton AB T6G 1Z2, Canada; irwindeep.sandhu@ualberta.ca; 3 Department of Family Medicine, University of Alberta, Edmonton, AB T6G 2T4, Canada

**Keywords:** multiple myeloma, lymphoproliferative disorders, survivorship

## Abstract

Multiple myeloma (MM) is a malignant clonal plasma cell disorder in the bone marrow and is the second-most common hematologic malignancy in adults. Although patients with MM have a moderate life expectancy, it remains a heterogeneous disease that often requires multiple lines of chemotherapy for durable control and long-term survival. This review outlines current management strategies for both transplant-eligible and transplant-ineligible patients as well as for relapsed and refractory disease. Advances in drug therapies have widened management options and improved survival. In this paper, we also discuss implications for special populations and survivorship care.

## 1. Introduction

Multiple myeloma (MM) is an incurable lymphoproliferative disorder of malignant clonal plasma cells originating in the bone marrow. It is the second-most common hematologic malignancy in adults, with a median age at diagnosis of 69 years [1]. According to the Canadian Cancer Society, it is estimated that 4000 Canadians will be diagnosed with MM in 2022 [2]. MM now represents 1.6% of new cancer diagnoses in Canada [2]. Both the incidence and prevalence of MM in Canada is on the rise due to improvements in case ascertainment rates, improved overall survival, as well as a growing, aging population [2]. The survival for patients with MM has improved substantially over the past 25 years largely due to advances in drug therapies, with novel drug classes now available as routine care [3].

In Canada, we have routine access to multiple drug classes including immunomodulatory agents (IMiDs), proteasome inhibitors (PIs), monoclonal antibodies directed against CD38 glycoprotein or SLAMF7 glycoprotein, and immunocellular therapies targeting several plasma cell antigens including B-cell maturation antigen (BCMA), G protein-coupled receptor, class C group 5 member D (GPRC5D), and the Fc receptor-like protein 5 (FCRL5) using various platforms including bispecifics, antibody-drug conjugates, and manufactured chimeric antigen receptor (CAR) T-cells [4,5,6]. With these new drug therapies, the median survival for patients with MM in Canada exceeds 5 years and, increasingly, many patients are living more than a decade beyond their initial diagnosis [7]. There is still no cure for MM, and most patients require continuous or serial treatments during the course of their lifetime. For these reasons, MM has transformed into a chronic disease for many patients. 

Beyond a focus on survival, patients who are living chronically with MM may prioritize quality of life, including the management of disease-related and treatment-related side effects, as well as the prevention, diagnosis, and management of other age-related conditions. Collaborative care with primary care providers is essential to provide balanced and well-rounded care for patients living with MM. Myeloma and cell therapy specific side effects are best treated by care providers who are familiar with the medications involved. Optimizing adverse treatment side effects improves the efficacy of anti-myeloma treatment [8]. The goals of patients with MM may be met through a close relationship with their primary care physician to address new symptoms and optimize comorbidities including cardiovascular and neuropsychiatric conditions. The need is present for generalists who are familiar with the latest information on cardiovascular event prevention, endocrinopathies such as diabetes and thyroid disorder, and second malignancy screening. The last point is of interest in an age-appropriate manner for patients, who now have a likelihood of living into the second decade with MM; have been exposed to potential carcinogenic chemotherapeutic agents such as cyclophosphamide, melphalan, and lenalidomide; and have immune dysfunction, in the surveillance for second malignancies [9]. The present manuscript intends to support the skillset of primary care physicians in reviewing the current anti-myeloma treatment paradigms in Canada (including individual drugs and drug combinations), outline common treatment- and disease-related toxicities, and outline common supportive treatment strategies. 

## 2. Diagnosis and Prognosis

### 2.1. Diagnosis

Patients with MM often present with typical but undifferentiated clinical features including fatigue, acute renal failure, hypercalcemia, normocytic anemia, weight loss, immunosuppression, or bone pain resulting in delays in diagnosis compared to other malignancies [10]. Generalists are at the frontline of cancer diagnosis and have the skills and knowledge to decrease diagnostic intervals [11]. The diagnosis of MM is based on the revised International Myeloma Working Group criteria [12]. It requires the demonstration of clonal bone marrow plasma cells > 10% or biopsy-proven plasmacytoma as well as one or more of the established CRAB (hypercalcemia, renal failure, anemia and bone lesions) clinical features and/or high-risk biomarkers (Table 1). A monoclonal protein is often detectable in the patient’s serum or urine by using specialized tests such as serum protein electrophoresis with immunofixation (SPEP/IFE), urine protein electrophoresis with immunofixation (UPEP/IFE), or serum free light chain assay. Patients with a monoclonal protein and clinical features or biomarkers of MM require urgent referral to a hematologist for confirmatory diagnosis and treatment.

### 2.2. Prognosis

Outcomes for patients with MM are known to be variable. The International Staging System (ISS) for MM was published in 2005 to provide a simple method using readily available lab tests, serum beta2-microglobulin, and serum albumin, to classify MM patients for treatment including experimental treatment [13]. Serum beta2-microglobulin and serum albumin strongly correlate with patient survival; however, the underlying reasons for this correlation is not completely understood [13]. The ISS classifies MM patients into three stages: Stage I—median survival 62 months, Stage II—median survival 44 months, and Stage III—median survival 29 months [13]. Although overall outcomes improved over the next decade, variability in outcomes remained wide. This variability called for a need to refine staging further for decision-making in an era of novel therapies. The International Myeloma Working Group published the Revised International Staging System (R-ISS) for Multiple Myeloma in 2015 to better classify patients for the purpose of personalizing treatment recommendations based on prognosis [14]. In addition to the ISS, the R-ISS includes chromosomal abnormalities determined by interphase fluorescent in situ hybridization (iFISH) and serum lactate dehydrogenase (LDH) to classify patients into three stages: Stage I, Stage II, and Stage III [14]. Five-year overall survival rates for R-ISS stage I, II, and III were 82%, 62%, and 40% respectively [14]. The R-ISS provides an improved staging system, but it continues to be limited by studies that only include patients under the age of 65 and are of short duration. Future iterations or new staging systems may need to consider additional factors including patient factors such as age, performance status, and comorbidities and include longitudinal studies of disease outcome. Use of geriatric modeling and frailty scores provides a clinical method to gauge prognosis, but needs further validation in prospective studies [15]. Lastly, modern genetic sequencing has provided a definition for ultra-high risk myeloma that helps to identify a population with survival less than 3 years [16,17].

## 3. Current Management

**Figure 1:**
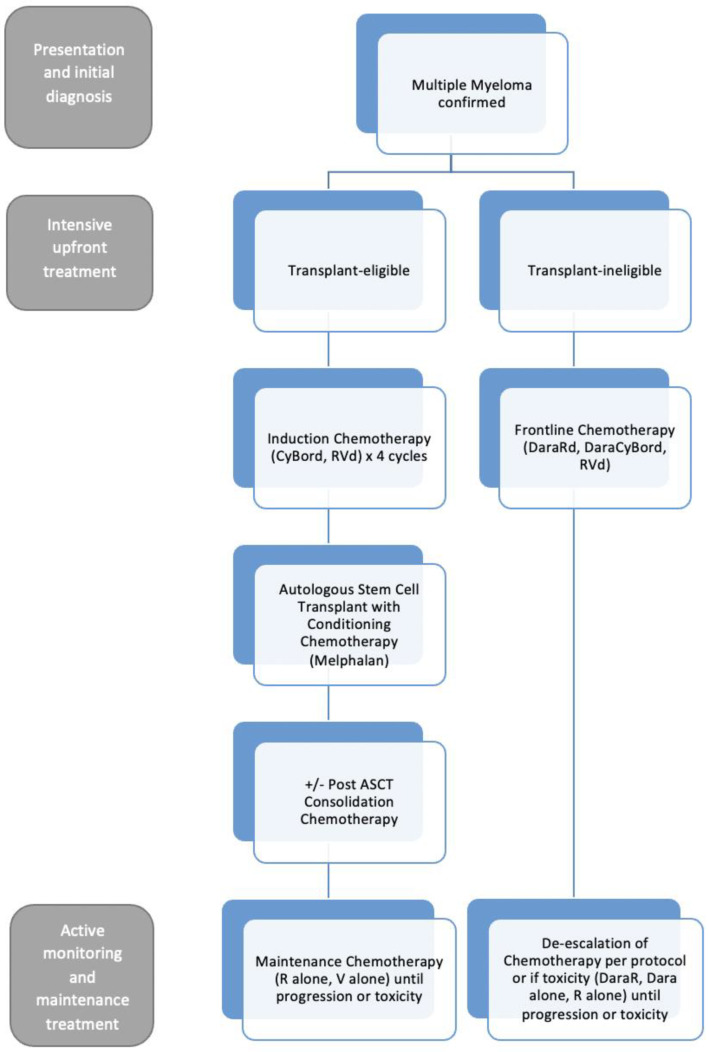
Frontline therapy for patients with newly diagnosed multiple myeloma in Canada. Abbreviations: CyBord, cyclophosphamide/bortezomib/dexamethasone; RVd, lenalidomide/bortezomib/dexamethasone; DaraRd, daratumumab/lenalidomide/dexamethasone; DaraCyBord, daratumumab/cyclophosphamide/bortezomib/dexamethasone; Rd, lenalidomide/dexamethasone; R alone, lenalidomide; V alone, bortezomib; Dara alone, daratumuab; MM, multiple myeloma; ASCT, autologous stem cell transplantation.

MM is an incurable lymphoproliferative disorder and requires sequential courses of treatment. When active MM is diagnosed, systemic treatment is almost always recommended. MM therapy has evolved several times over the last 25 years, now with numerous drug classes available for the treatment of patients with MM in Canada. A summary of common anti-myeloma pharmacotherapy is listed in Table 2 and a summary of drug-related side effects is listed in Table 3. Patients are initially considered as two groups—those who are transplant eligible (e.g., age < 65–70 years, fit, few comorbidities) and those who are transplant ineligible (e.g., age > 65–70 years, numerous or significant comorbidities, frail, unfit). Attempts to define these treatment pathways have been documented, but may become outdated quickly given the rapid pace of innovation in this [18]. Figure 1 outlines an approach to the management of patients with newly diagnosed MM.

### 3.1. Transplant Eligible

Patients who are eligible for possible autologous stem cell transplant (ASCT) undergo induction chemotherapy with a triplet drug regimen, commonly cyclophosphamide, bortezomib, dexamethasone (CyBorD) or lenalidomide, bortezomib, and dexamethasone (RVD) in Canada. Many patients receive induction chemotherapy for four cycles (each cycle is 4 weeks in length). Subsequently, patients undergo autologous bone marrow stem cell apheresis or collection by peripheral blood and then receive conditioning chemotherapy with high dose melphalan followed by ASCT. After recovering from their ASCT, patients may start consolidation with RVD followed by maintenance chemotherapy with oral lenalidomide (most common) or subcutaneous bortezomib injections (occasionally). Patients continue maintenance therapy until the time of drug intolerance or disease progression, whichever occurs first. At the time of relapse, patients will start a new chemotherapy treatment, often a triplet or three-drug containing regimen.

### 3.2. Transplant Ineligible

Patients who are transplant ineligible will receive initial treatment with a triplet drug regimen. In many provinces in 2023, the recommended frontline treatment would be a daratumumab-based regimen, often with the combination of lenalidomide and dexamethasone. Very elderly or frail transplant-ineligible patients may receive a dose-reduced doublet regimen only given the trend of therapy side effects leading to drug holidays and increased mortality [15]. Patients continue the daratumumab and lenalidomide component of their treatment until the time of drug intolerance or disease progression, whereas the bortezomib and dexamethasone components of the regimens are completed after 6 to 12 months. 

### 3.3. Relapsed or Refractory Disease

**Figure 2:**
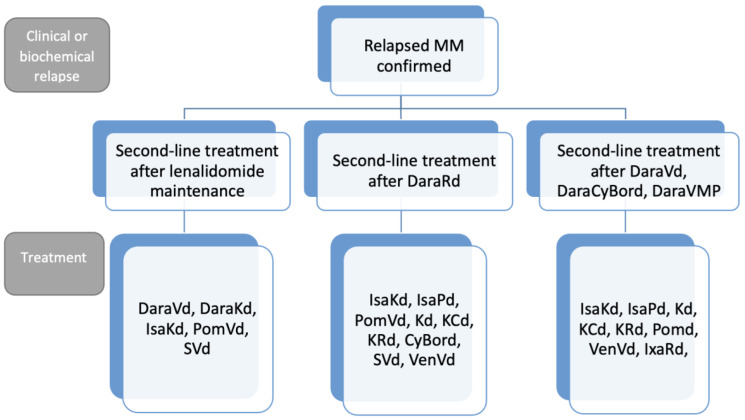
An approach to the management of patients with relapsed/refractory multiple myeloma.

The treatment of relapsed/refractory MM (RRMM) is complex and depends on numerous elements including disease characteristics, patient factors (such as age, frailty, ability to attend weekly for injection treatment), treatment history, drug-class refractory status, and the availability of clinical trials. Triplet regimens are often preferred over doublet regimens. Figure 2 outlines an approach to the management of patients with RRMM.

### 3.4. Monitoring of Disease

Monitoring disease status commonly includes the use of monthly blood and urine tests. The serum protein electrophoresis, which looks for the heavy and light chain of the abnormal monoclonal antibody, can be used for most patients to monitor for disease relapse. The monoclonal protein value is used as a surrogate for disease bulk. Urine protein electrophoresis are also standard, but the use of 24 h collections versus spot samples is a question that remains to be defined. The standard criteria would require a 24 h collection, but many patients find it cumbersome to properly collect and to adequately transport. The added value of the 24 h collection is uncertain in the era of serum-free light chain assay [19,20]. The benefit of maintaining the urine monitoring would be to look for proteinuria secondary to medications such as bisphosphonates or to rule out subsequent development of AL amyloidosis. The serum free light chain assay itself should be used monthly to help monitor for serum-free light chain escape, which is an evolution of the tumour from producing both heavy and light chains to that of only light chains. This phenomenon occurs in 10% of patients [21]. Quantitative immunoglobulins is the last test used for IgA myeloma to help define disease relapse [22]. The testing looks for biochemical progression, which is the growth in the abnormal protein beyond a minimal threshold, for example, the doubling of the monoclonal protein number with a minimal increase in 5 g/L. Criteria for progression is defined using the international uniform response criteria [23]. All signs of progressive disease must be remeasured with a second set of labs tests performed no later than the start of the subsequent cycle.

### 3.5. Special Populations

#### 3.5.1. Older Adults

Although we have seen significant improvements in disease-free progression and overall survival among patients over the past decade, variable outcomes remain in the elderly population because of greater heterogeneity within this population [24]. Empirical studies have shown that as individuals age, heterogeneity increases in physical performance measures, chronic conditions, and frailty [25]. For this reason, chronological age alone cannot be used to determine initial treatment and treatment of relapsed or refractory disease; otherwise, there may be a risk of undertreating those who are fit for treatment and overtreating those who are frail and at higher risk of treatment toxicities [26,27].

Tailoring treatment among older adults remains an area of ongoing research. A better understanding of the heterogeneity of this population through inclusion of clinically relevant variables such as fitness and frailty assessments in trials is essential to optimize and offer treatment based on biological rather than chronological age [26,27]. At present, it is important that all patients regardless of chronological age should be given the opportunity for assessment and consideration of treatment options using emerging fitness and frailty assessment tools [28,29].

Finally, recent studies on optimization and maintenance of fitness in MM patients have demonstrated improvement in both physical and psychological outcomes [30,31]. Traditionally, there has been a focus on rehabilitation following cancer treatment; however, newer studies are investigating the benefits of prehabilitation where assessment and interventions prior to the start of cancer treatment can improve outcomes [32]. For MM, where treatment may be continuous or involve serial treatments, development of strategies involving a multidisciplinary team for maintenance of fitness is particularly warranted in this population.

#### 3.5.2. Younger Adults

MM is considered a disease of the elderly, but the reality is that up to 40% of newly diagnosed MM are in patients under 65, with approximately 15% in patients under 55 [1,33]. Currently, most clinical trials involve patients who are over 55, which limits the generalizability of the results to younger adults [34]. In addition, there is limited knowledge about the long-term efficacy of treatment regimens within this population. However, perhaps more challenging in younger adults are the psychological and physical aspects of living with cancer and their impacts on work productivity and quality of life [35,36].

Qualitative studies and reviews have shown that in addition to treatment, patients value the ability to maintain daily activities and are concerned by the financial consequences of living with MM [37,38,39,40]. Although continuing activities of daily living and maintenance of financial well-being are important for all patients, they are particularly relevant to younger adults who are often still a part of the workforce, and have children to support. More studies are needed to understand the relationship between management of MM and patient-reported outcomes as well as how to deliver a multidisciplinary team approach to management that involves many different health professionals. In the meantime, primary care physicians play an important role in survivorship and coordinating care.

## 4. Survivorship

Although MM is incurable, continuous or serial therapy has transformed it into a chronic disease [41]. Therefore, in addition to the traditional focus of survival, health care professionals have an increasingly important role in supportive care, with a focus on quality of life including prevention of both disease- and treatment-related complications [42,43]. Patients with MM often show symptoms to their primary care physician that may or may not be disease- or treatment-related. Knowing disease- and treatment-related presentations will help primary care physicians provide survivorship support for MM, as seen Table 4.

### 4.1. Bone Disease

Bone disease is a hallmark of MM, affecting up to 80% of newly diagnosed patients and often associated with pain [44,45]. In addition, more than 60% of patients will develop a fracture during the course of the disease because of multiple disruptions to normal bone metabolism induced by MM through dysregulation of the normal bone remodeling process involving osteoclasts, osteoblasts, osteocytes, bone matrix, and immune cells [40,45,46,47]. In a population-based study, patients with a fracture either at diagnosis or during the course of their disease were at a significantly increased risk of death [46]. As a result, prevention of bone disease and fractures is a priority. 

Patients may see their primary care physician with an array of presentations for their bone disease including bony pain, vertebral compression fractures, and osteoporosis. In addition, incidental lesions may be seen on imaging. In MM, bony lesions are classically lytic lesions as a result of destruction of the bone as opposed to blastic or sclerotic lesions caused by new bone formation. Lytic lesions are best detected using whole body low dose computerized tomography (CT) because of its higher sensitivity in identifying lytic bone disease and detecting extra-osseous disease compared to conventional X-rays [48,49]. However, conventional radiographic skeletal surveys can continue to be considered based on patient factors and local resources (availability and cost). When lytic lesions are detected, management considerations include localized radiation or vertebroplasty for symptom control, surgical intervention for impending fractures, and bone protectors such as bisphosphonates to prevent further progression of bone disease [45].

Hypercalcemia because of impaired bone metabolism is commonly detected through abnormal lab investigations; however, patients may present the classic symptoms of depressed mood, bony pain, renal colic, and abdominal pain. Management revolves around hydration, discontinuation of vitamin D and calcium supplementation, treating the underlying MM disease, and consideration of a bone protector [45]. In this setting, intravenous (IV) bisphosphonates such as pamidronate and zoledronic acid are favoured over oral preparations, with a 5.5 month survival advantage noted in a phase 3 trial designed to answer that question [50,51].

Low bone density or osteoporosis may be detected in patients, and although the use of bone protectors in these patients without lytic lesions is less clear, it may be an option that requires shared decision-making between a physician and the individual patient. An important factor to consider when recommending bisphosphonates is the risk of osteonecrosis of the jaw (ONJ), which can occur in up to 11% of patients [45,52]. The risk of ONJ is higher with the use of IV bisphosphonates, invasive dental procedures, and the long-term use of bisphosphonates in MM patients because of improved survival [52]. As a part of supportive care, proper dental hygiene and regular dental care may help mitigate this risk [45].

Avascular necrosis of the femoral head is a complication of dexamethasone-based regimens. The overall incidence of this in MM patients treated with dexamethasone is 6.7% [53]. Although the mechanism of this complication is unknown, it is thought to be related to dose and duration of dexamethasone use. Symptoms typically do not occur until the avascular necrosis is advanced; thus, involvement of the orthopedic surgeon should be considered [45].

### 4.2. Renal Disease

Renal disease is common in MM with up to 50% of patients having renal insufficiency at diagnosis [54]. Although it is unknown what proportion of renal disease is directly due to MM, MM-related renal disease is thought to be caused by monoclonal light chains damaging the kidneys. Multiple mechanisms may be involved including but not limited to monoclonal light chains obstructing renal tubules and deposition of monoclonal immunoglobulins (Igs) on glomerular or tubular basement membranes [54]. In addition, there are Ig-independent causes of renal disease in MM patients including volume depletion, hypercalcemia, hyperuricemia, nephrotoxic drugs, and infection, leading to a variety of nonspecific symptoms such as fatigue, pruritus, pain, decreased mood, decreased appetite, and sleep disturbances. Despite the variable presentation and mechanism of renal disease in MM, prognosis is often dependent on response to treatment of the underlying MM [54]. 

Given the prevalence of renal disease in patients living with MM and the unknown contribution of Ig-mediated versus Ig-independent renal disease, approach to renal disease for the primary care physician includes appropriate hydration, management of hypercalcemia and hyperuricemia, avoidance of nephrotoxic drugs such as non-steroidal anti-inflammatories, and consideration of involvement of nephrology if renal functioning continues to decline [42,43,55]. This ensures that the chronic renal insufficiency effects on the body such as hypertension, hypercalcemia, low vitamin D levels, the need for erythropoietin stimulating agents, and the preservation of renal function are monitored and align with other chronic kidney disease populations.

### 4.3. Infections

Infection is a significant cause of morbidity and mortality for patients with MM. Research has shown that disease-related factors such as MM-induced neutropenia and treatment-related immunosuppression confer a 10-fold and 7-fold increased risk of viral and bacterial infections, respectively [56]. Patients with MM are at highest risk of infection during the first 6 months of diagnosis including up to 22% of patients with infection as the underlying cause of death during the first year of diagnosis [56]. Prophylactic antibiotics and antivirals, inactivated vaccines, and immunoglobulin replacement may mitigate infections [57]. Prophylactic fluoroquinolone may be prescribed by the specialist team within the first 3 months of diagnosis for patients, beginning active treatment due to a peak in infections during induction therapy. Prophylactic antivirals (e.g., acyclovir, valacyclovir) are routinely prescribed by the specialist team for patients receiving proteasome inhibitors such as bortezomib and monoclonal antibodies. Additionally, patients receive antivirals after autologous stem cell transplant. Despite the lack of efficacy trials and potentially limited response, inactivated vaccines remain the mainstay of prevention due to safety and potential benefits of partial response prior to initiation of treatment of MM, while inactivated influenza, pneumococcal polysaccharide and COVID vaccines should be administered and updated for all patients throughout the course of disease. Patients with proven symptomatic influenza and COVID should be treated according to current public health recommendations. In patients using modern anti-myeloma therapy such as CAR T-cell and bispecific therapies, hypogammaglobinemia is increasingly seen. A history of hypogammaglobinemia with severe or recurrent infections, and immunoglobulin replacement, with or without long-term antibiotics, may be considered [57]. Vaccination schedules post-stem-cell transplant and CAR T-cell therapy are listed separately by provinces, and should be individualized based on provincial approval and current therapy for the patient’s myeloma. Novel treatments such as bispecific T cell engagers and CAR T-cell therapy are associated with higher rates of atypical infections, such as Pneumocystis jiroveci pneumonia (PJP) and Cytomegalovirus (CMV) infections. Having a heightened awareness for this T-cell dysfunction and the subsequent infections is key to early diagnosis and consultation with infectious disease specialists.

### 4.4. Thrombosis

The cause of thrombosis, both venous and arterial, in patients with MM is multifactorial. Disease-related factors include tumor load and inflammatory cytokines, treatment-related factors include drug effects, particularly immunomodulatory drugs, on coagulation factors and platelet aggregation while patient-related factors include personal risk factors and genetic predisposition [58]. For high-risk patients, low molecular weight heparin (LMWH) and warfarin remain the management of choice. For lower-risk patients, acetylsalicylic acid (ASA) is advised. Currently, the role of direct oral anticoagulants (DOACs) is being investigated in cancer-associated thrombosis and is an emerging option; however, it is important to note that many studies have a smaller number of participants with MM compared to other malignancies [58]. In addition, many of the studies focused on venous thrombosis rather than arterial thrombosis.

### 4.5. Second Malignancy

Improved prognosis for MM translates into a higher risk of developing a second malignancy. The rate of a second malignancy has been estimated to be between 5 and 7% with the incidence higher for a second hematological malignancy compared with a second solid organ malignancy [59]. There are high rates of second solid organ malignancies, with the highest risk for non-melanotic skin malignancies, followed by high rates of gastrointestinal, kidney, bladder, and melanoma malignancies [59]. There are multiple factors for the increased risk of all second malignancies which can be classified into disease-related, treatment-related, and patient-related factors. Disease-related factors include genetics and immune dysregulation, treatment-related factors include different drug effects and duration of treatment, and patient-related factors include age, sex, genetic predisposition, and environmental risks [59]. Awareness of risk and types of second malignancies is important in considering next steps when patients present with a concern such as a new skin lesion where a low threshold for biopsy is needed [60].

### 4.6. Peripheral Neuropathy

Up to 20% of MM patients have peripheral neuropathy (PN) at diagnosis, which has been postulated to be a result of the monoclonal protein or direct nerve compression, while as many as 75% may experience PN during therapy as a result of treatment [61,62]. Incidence of treatment-induced PN is dependent on treatment agent and treatment combination. Although treatment-induced PN may be dose- or duration-related, there is tension between dose reduction in the management of side effects and dose maintenance for disease control, as well as tension between alteration of treatment duration to prevent permanent neurological damage and maintenance of duration to prevent relapse of disease [61].

Management of PN commonly involves anti-epileptic agents and antidepressants. Although recent studies have investigated topical baclofen, amitriptyline and ketamine, venlafaxine, topical menthol cream, and electro-acupuncture, there are limited studies involving MM patients. [62].

### 4.7. Gastrointestinal Effects

Diarrhea is one of the most common gastrointestinal side effects of treatment of MM. When alternative etiologies for diarrhea such as infectious or inflammatory are ruled out, diarrhea due to anti-myeloma treatment is often managed conservatively through adequate hydration and diet modification. Although medications such as loperamide and ondansetron can be effective anti-diarrheals, bile acid sequestrants should be considered because anti-myeloma treatment can increase biliary acid secretion [43]. With new agents such as bispecific T-cell engager and CAR t-cell therapy emerging, atypical infections such as CMV need to be considered.

### 4.8. Cardiotoxicity

Since MM is a disease of the elderly, many patients have pre-existing cardiovascular disease [63]. However, it is important to be aware that IMiDs and PIs are associated with cardiotoxicities such as heart failure, hypertension, arrhythmia, and coronary artery disease, particularly during the early stages of treatment [64]. In a recent network meta-analysis, the use of IMiDs in MM patients leads to a two-fold higher risk of cardiotoxicity compared to untreated MM patients [65]. In addition, the authors report that the cardiotoxic risk for PIs may not be class-specific, with carfilzomib demonstrating the most significant cardiotoxicity [65]. Understanding the cardiotoxic potential of certain treatment regimens increases vigilance for identifying and managing cardiovascular complications in MM patients.

## 5. Conclusions

Management of MM has changed in Canada with the approval of novel therapies. As a result, there is improved survival. However, gaps remain in optimization of therapy particularly for the older adult. As tools are applied to better tailor treatment, survivorship and supportive management of complications and treatment-toxicities of MM will also become increasingly important.

### Key Points

- Novel therapies have increased treatment options and improved prognosis for MM.
- The assessment of fitness and frailty is increasingly being evaluated as a way of personalizing treatment for MM.
- The main treatment toxicities are infection, renal disease, peripheral neuropathy, diarrhea, and bone disease.
- Long-term therapy has changed MM into a chronic disease.
- Supportive care involving multidisciplinary teams to manage disease-, treatment- and patient-related complications is increasingly important.

## Figures and Tables

**Table 1:** Diagnosis of Multiple Myeloma—Revised International Myeloma Working Group (IMWG) Criteria.

Diagnosis	Criteria			
Smoldering Multiple Myeloma	Both criteria required	1	No myeloma–defining events or amyloidosis	
		2		Clonal bone marrow plasma cells 10–60%
			OR	Serum monoclonal protein (IgG or IgA) ≥ 30 g/L
			OR	Urinary monoclonal protein ≥ 500 mg per 24 h collection
Active Multiple Myeloma	Both criteria required	1	Clonal bone marrow plasma cells ≥ 10% or biopsy-proven bony or extramedullary plasmacytoma	
		2	Any one or more of the following myeloma-defining events (MDEs) which include clinical CRAB features or biomarkers of malignancy	Clinical CRAB features Hypercalcemia: serum calcium > 0.25 mmol/L higher than the upper limit of normal or >2.75 mmol/L Renal insufficiency: creatinine clearance < 40 mL per minute or serum creatinine > 177 mol/L Anemia: hemoglobin value of >20 g/L below the lowest limit of normal, or a hemoglobin value < 100 g/L Bone lesions: one or more osteolytic lesions on skeletal radiography, CT, or PET-CT. If bone marrow has <10% clonal plasma cells, more than one bone lesion is required to distinguish from solitary plasmacytoma with minimal marrow involvement. Biomarkers of malignancy 60% or greater clonal plasma cells on bone marrow examination Serum involved/uninvolved free light chain ratio of 100 or greater, provided the absolute level of the involved light chain is at least 100 mg/L More than one focal lesion in bone marrow on MRI that is at least 5 mm or greater in size.

Adapted with permission from Ref. [12]. 2014, Lancet Oncology.

**Table 2:** Common anti-myeloma pharmacotherapy.

Drug Class	Generic Drug Name (Trade Name)	Route of Administration	Abbreviation	Common Regimens Used in Canada
Immunomodulatory drugs (IMiDs)				
	Thalidomide (Thalomid)	PO	T	TD
	Lenalidomide (Revlimid)	PO	R	RD, RVD, DRD, KRD, IxaRD
	Pomalidomide (Pomalyst)	PO	P or Pom	IsaPD, PD,
	Iberdomide (CC220)	PO	Iber	Being evaluated in clinical trials
	Mezigdomide (CC92480)	PO	Mezi	Being evaluated in clinical trials
Proteasome Inhibitors (PIs)				
	Bortezomib (Velcade), B or V	SC, IV	B or V	CyBorD/VCD, Dara- CyBorD/Dara-VCD, DVD,
	Carfilzomib (Krypolis), C or K	IV	C or K	KRD, KCD
	Ixazomib (Ninlaro), Ixa	PO	Ixa	IxaRD, Ixa
Monoclonal Antibodies				
	Daratumumab (Darzalex)	SC, IV	D or Dara	DRD, DVD
	Isatuximab (Sarclisa)	IV	Isa	IsaPD, IsaKD
	Elotuzumab (Empliciti)		Elo	(not approved in Canada)
Immunoconjugates				
Antibody-drug conjugate	Belantamab mafodotin/(Blenrep)	IV	Bela or Belamaf	Bela monotherapy (Health Canada approved; not routinely funded)
Bispecific antibody	Elranatamab,	SC		Being evaluated in clinical trials
	Teclistamab	SC		Being evaluated in clinical trials
Other novel mechanisms				
	Selinexor (Xpovio)	PO	S or Seli	SD, SVD/SBD
	Venetoclax (Venclexta)	PO	Ven	VenD, VenVD/VenBD
chimeric antigen receptor (CAR) T-cell				
	idecabtagene vicleucel (bb2121)	IV	ide-cel	Being evaluated in clinical trials; pending regulatory approval and CADTH review
Alkylating agents				
	Melphalan (Alkeran)	PO, IV	M	BMP/VMP
	Cyclophosphamide (Cytoxan)	PO, IV	Cy	CyBorD/VCD, Dara-CyBorD/Dara-CVD
Steroids				
	Dexamethasone	PO, IV	D	Numerous combinations per this table
	Prednisone	PO	P	CycloPred, BMP/VMP

**Table 3:** Summary of drug-related side effects, by specific drug (alphabetical). Data from Ref. [4]. 2023, Cancer Care Ontario; Ref. [5]. 2023, BC Cancer Agency; Ref. [6]. 2023, CADTH.

Generic Drug Name (Trade Name)	Common Toxicity (>25%)	Less common Toxicity (2–25%)	Rare Toxicity (<1%)
Belantamab mafodotin/Belamaf (Blenrep)	Keratitis, keratopathy, dry eye, infections, anemia, thrombocytopenia, nausea, constitutional symptoms, fever, AST increase	Bleeding, constipation, vomiting, pneumonia, sepsis, infusion related reaction, elevated liver enzymes, renal failure, hypercalcemia	Not reported
Bortezomib (Velcade)	Infection (including viral reactivation), thrombocytopenia, bleeding, neuropathy (sensory, motor, autonomic), rash, nausea, diarrhea, constipation, anorexia, fatigue, headache	Dyspepsia, peripheral edema, electrolyte abnormalities, insomnia, myalgias, dizziness, cough, blurred vision	Hypertension, posterior reversible encephalopathy syndrome (PRES), congestive heart failure (CHF), acute liver failure, pneumonitis
Carfilzomib (Krypolis)	Infection (including viral reactivation), thrombocytopenia, bleeding, hypertension, diarrhea, nausea, vomiting, edema, fatigue, infusion related reaction, cough, dyspnea, insomnia, myalgias	Venous and arterial thromboembolism, cardiotoxicity, pulmonary hypertension, abdominal pain, dyspepsia, increased liver enzymes, abnormal electrolytes, renal failure, weakness, headaches, peripheral neuropathy, Acute respiratory distress syndrome (ARDS)	Thrombotic microangiopathy (TMA), PRES, Progressive multifocal leukoencephalopathy (PML), CHF
Cyclophosphamide (Cytoxan)	Myelosuppression, infections, bleeding, alopecia, nausea, vomiting	Fatigue, increased liver enzymes, myalgias, hemorrhagic cystitis	Arrhythmia, arterial and venous thromboembolism, cardio toxicity, pancreatitis, vaso-occlusive disease, rash, hypersensitivity including anaphylaxis, rhabdomyolysis, second malignancy, syndrome of inappropriate antidiuretic hormone (SIAHD), neurotoxicity, PRES, nephrotoxicity, pneumonitis, vasculitis
Daratumumab (Darzalex)	Infusion related reaction	Infections, bleeding, anemia, hypertension, constipation, diarrhea, nausea, vomiting, edema, fatigue, increased liver enzymes, abnormal electrolytes, myalgias, headaches, insomnia, cough, dyspnea, rhinitis	Atrial fibrillation, cardiotoxicity, pancreatitis, cytokine release syndrome (CRS), anaphylaxis
Dexamethasone (Decadron)	Peripheral edema, gastrointestinal upset, nausea, gastroesophageal reflux disease (GERD), insomnia, mood disturbances	Gastritis, hyperglycemia, infection, increased appetite, weight gain, hypertension, hyponatremia, hypokalemia, delayed wound healing, cataracts, skin changes, adrenal suppression, proximal muscle weakness, osteoporosis	Peptic ulcer, perforation, avascular necrosis of the femoral head, seizures, psychosis
Isatuximab (Sarclisa)	Infusion related reaction, myelosuppression, infection, anemia, neutropenia, thrombocytopenia, pneumonia, upper respiratory tract infection, diarrhea	Arrhythmia, atrial fibrillation, nausea, stomatitis, vomiting, edema, bronchitis, herpes viral infection, nasopharyngitis, arthralgias, weakness, myalgias, second malignancy, headache	Not reported
Ixazomib (Ninlaro)	Infection (including viral reactivation), thrombocytopenia, bleeding, diarrhea, constipation, nausea, vomiting, peripheral neuropathy, edema, fatigue, infusion related reaction, cough, dyspnea, insomnia, ocular disorders,	Rash, pruritis, anorexia, myalgias, hypokalemia, dizziness, headache,	Thrombotic thrombocytopenia purpura (TTP), hepatotoxicity, Steven-Johnson syndrome (SJS), Sweet’s syndrome, toxic epidermal necrolysis (TEN), Drug rash with eosinophilia and systemic symptoms (DRESS)
Lenalidomide (Revlimid)	Nausea, vomiting, diarrhea, constipation, infection, bleeding, edema, fatigue, myalgias, headache, cough, dyspnea	Rash, arrythmia, venous and arterial thromboembolism, HTN, abdominal pain, anorexia, peripheral neuropathy, depression, syncope, tremor, blurred visions, abnormal electrolytes, thyroid disease	SJS, TEN, DRESS, cholecystitis, pancreatitis
Melphalan (Alkeran)	Nausea, diarrhea, myelosuppression, infection, anemia, neutropenia, injection site reaction, flushing, rash, alopecia	Mucositis, esophagitis, vomiting, diarrhea, tumour lysis syndrome, hepatitis, infertility, amenorrhea, azoospermia, renal failure, second hematologic and solid organ malignancies	Anaphylaxis, tissue necrosis (if extravasation), pneumonitis, pulmonary fibrosis
Pomalidomide (Pomalyst)	Myelosuppression, infection, bleeding, diarrhea, fatigue	Atrial fibrillation, rash, anorexia, Constipation, diarrhea, nausea, vomiting, edema, increased liver enzymes, abnormal electrolytes, myalgias, dizziness, headache, insomnia, peripheral neuropathy, cough, dyspnea	Hypertension, venous thromboembolism second skin malignancies, pneumonitis, interstitial lung disease, tumor lysis syndrome, severe hypersensitivity reaction including SJS, TEN, DRESS, hepatic failure
Prednisone	Peripheral edema, GI upset, nausea, GERD, insomnia, mood disturbances	Gastritis, hyperglycemia, infection, increased appetite, weight gain, hypertension, hyponatremia, hypokalemia, delayed wound healing, cataracts, skin changes, adrenal suppression, proximal muscle weakness, osteoporosis	Peptic ulcer, gastrointestinal perforation, avascular necrosis of the femoral head, seizures, psychosis
Selinexor	Nausea, diarrhea, constipation, fatigue, decreased appetite, myelosuppression, infection, bleeding, thrombocytopenia, anemia,	Vomiting, weight loss, dehydration, renal failure, hyponatremia, hypokalemia, visual disturbances, dizziness, dyspnea, and upper respiratory tract infection, confusion, altered mental status	Arrhythmia, birth defects
Venetoclax	Myelosuppression, infection, neutropenia, bleeding, diarrhea, nausea, fatigue, myalgias	Rash, pruritus, abdominal pain, constipation, vomiting, edema, hemolytic anemia, abnormal electrolytes, tumour lysis syndrome, second malignancies, dizziness, headache, cough	Cholecystitis, renal failure

**Table 4:** Supportive management of common complications of multiple myeloma.

**Complication**		**Disease Factors**	**Treatment Factors**	**Patient Factors**	**Supportive Management**
Bone disease	Lytic lesions	Dysregulation of normal bone metabolism Osteoclastic activity			Pain management Steroid Radiation Kyphoplasty Vertebroplasty Orthopedic referral
	Hypercalcemia	Dysregulation of normal bone metabolism Osteoclastic activity Ig-dependent renal disease	Denosumab	Age Nutrition Gender Comorbid disease Non-antimyeloma drugs	Hydration Discontinue calcium and vitamin D Bisphosphonates
	Osteoporosis	Dysregulation of normal bone metabolism Osteoclastic activity			Bisphosphonates
	Osteonecrosis of the jaw		Bisphosphonates		Dental hygiene Dental care Dentist referral (specialist in oral and maxillofacial dentistry if available)
	Avascular necrosis		Steroids		Orthopedic referral Pain management
Renal disease	Ig-dependent	Cast nephropathy Monoclonal Ig deposition Light chain amyloidosis			Treatment dose modification Hydration Avoid nephrotoxic drugs Nephrology referral
	Ig-independent	Hypercalcemia	Carfilzomib Bisphosphonates	Age Nutrition Comorbid disease Non-antimyeloma drugs	
Infection	Viral	Immunoparesis (low immmunoglobulin levels, hypogammaglobulinemia) Impaired lymphocyte function	Multiple agents causing neutropenia PIs IMiDs Monoclonal antibodies	Age Nutrition Comorbid disease Non-antimyeloma drugs	Prophylactic antivirals Vaccination with inactivated vaccines
	Bacterial	Immunoparesis (low immmunoglobulin levels, hypogammaglobulinemia) Impaired lymphocyte function Neutropenia Hyposplenism	Melphalan Steroids Bispecific T-cell engagers CAR T-cell therapy		Prophylactic antibiotics Vaccination with inactivated vaccines
Thrombosis	Venous thrombo- embolisms Arterial thrombo- embolisms	Tumor load Inflammatory cytokines	IMiDs Dexamethasone	Comorbid disease Non-antimyeloma drugs	Low molecular weight heparin (LMWH) Warfarin Acetylsalicylic acid
Second malignancies	Hematological	Genetics Immune dysregulation	Alkylator therapy Lenalidomide Radiation	Age Genetics Comorbid disease Environmental exposures Non-antimyeloma drugs Alcohol use disorder Smoking history	
	Solid organ				Age-appropriate cancer screening Low-threshold for biopsy
Peripheral neuropathy	Sensory	Monoclonal proteins Direct compression	Bortezomib Thalidomide Melphalan Bispecific T-cell engagers	Diabetes mellitus Alcohol use disorder Vitamin deficiencies Viral infections Non-antimyeloma drugs	Antiepileptics Antidepressants
	Motor				
Gastrointestinal effects	Diarrhea		Short-term: Bortezomib, Panobinostat Late-onset: Lenalidomide	Comorbid disease Non-antimyeloma drugs	Treatment dose modification Dietary changes Loperamide Cholestyramine
Cardiac toxicity	Congestive heart failure Arrhythmia	Amyloid light-chain immunoglobulin deposition	Carfilzomib	Age Gender Comorbid disease	Cardiovascular risk management Treatment dose modification Diuretics Cardiology referral
